# A Multi-User Public Key Encryption with Multi-Keyword Search out of Bilinear Pairings

**DOI:** 10.3390/s20236962

**Published:** 2020-12-05

**Authors:** Shuo Zhang, Qiaoyan Wen, Wenmin Li, Hua Zhang, Zhengping Jin

**Affiliations:** State Key Laboratory of Networking and Switching Technology, Beijing University of Posts and Telecommunications, Beijing 100876, China; shuozhang@bupt.edu.cn (S.Z.); wqy@bupt.edu.cn (Q.W.); zhanghua_288@bupt.edu.cn (H.Z.); zhpjin@bupt.edu.cn (Z.J.)

**Keywords:** PEKS, public key encryption with keyword search, multi-keyword, multi-user

## Abstract

Internet of Things (IoT) and cloud computing are adopted widely in daily life and industrial production. Sensors of IoT equipment gather personal, sensitive and important data, which is stored in a cloud server. The cloud helps users to save cost and collaborate. However, the privacy of data is also at risk. Public-key encryption with keyword search (PEKS) is convenient for users to use the data without leaking privacy. In this article, we give a scheme of PEKS for a multi-user to realize the multi-keyword search at once and extend it to show a rank based on keywords match. The receiver can finish the search by himself or herself. With private cloud and server cloud, most users’ computing can be outsourced. Moreover, the PEKS can be transferred to a multi-user model in which the private cloud is used to manage receivers and outsource. The store cloud and the private cloud both obtain nothing with the keyword information. Then our IoT devices can easily run these protocols. As we do not use any pairing operations, the scheme is under more general assumptions that means the devices do not need to take on the heavy task of calculating pairing.

## 1. Introduction

**Figure 1:**
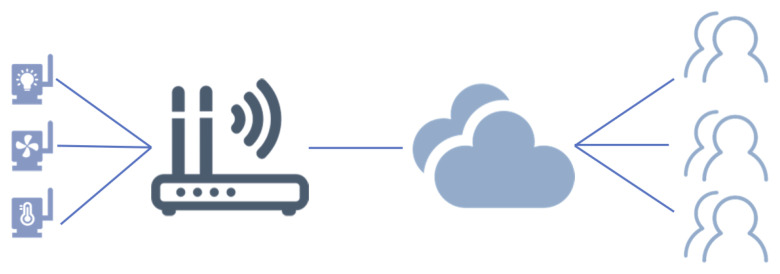
Internet of things.

The Internet of Things (IoT) devices have been used widely. Many people use a third-party cloud service to manage IoT devices (Figure 1). Through sensors, IoT devices monitor the environment in our homes or work place. So that we can get information from home or work place and make corresponding actions. Many of the sensors generate data that lives in the cloud. Personal, sensitive and important data which is stored in the cloud server. The cloud helps users to save cost and collaborate. The data contains a lot of private information that is at risk of being leaked. As perplexed by leakage of information, more people realize that the privacy is a big challenge in an era of big data.

Security protocols and cryptography tools are put forward to balance the privacy and convenience. For example, the chaotic image encryption is used to protect privacy [1]. Particularly, in order to realize more functionality, a specific security protocol is proposed against the threat of privacy leaks. Retrieval for encrypted data is significant for applied cloud storage. Storage outsourcing, private data sharing, mail routing and so on need a searchable encryption scheme which means retrieving keywords in encrypted data. To implement such function, private information is stored in encrypted form and calculation is finished without unfolding any confidential information.

In related years, searchable encryption has been paid more attention in the field of information security. The encrypted data stored in cloud is searchable and privacy is preserved. Two kinds of means are widely discussed in recent years which are called public-key encryption with keyword search (PEKS) and searchable symmetrical encryption (SSE). Both of them are focused on keyword search. Encryption protects the confidentiality of data and search feature makes it easy to employ the data. Private information retrieval (PIR) is also an analogous research direction which contains keywords and indexes as well. All of them help people structure data and find out what people want from encrypted data.

Homomorphic encryption is a natural tool for designing a functionality with unfolding encrypted data. A cloud with more computing power can help users calculate a searchable circuit or well designed string matching boolean function. However, fully homomorphic encryption still evolves quickly but is not enough efficient right now. For practical purposes, fully homomorphic encryption can not directly structure a feasible scheme. The homomorphism of algebra system or encryption scheme is one of most suitable character to design a searchable encryption. Bilinear pairings is widely used in PEKS. However, it is considered more expensive and higher-demand assumption than the traditional tools without pairings.

The definition of private information retrieval was first proposed by Chor, et al. [2] in 1995. The definition of public-key encryption with keywords search was first proposed by Boneh, et al. [3]. Baek et al. proposed a revisited scheme against the off-line keyword guess attack [4]. There are many research works related with PEKS. Fang, et al. [5] proposed a PEKS without random oracle. Xu, et al. [6] proposed a method with fuzzy keyword search. Yu et al. [7] realized a scheme with revocable keyword search. Chen et al. [8] presented a secure server-designation PEKS without a secure channel. Liu et al. [9] proposed a verifiable scheme based on key policy attribute-based encryption. Chen et al. [10] gave a scheme with dual-server to adapt cloud storage. Wu et al. [11] designed a lattice-based scheme in multiuser environments. Chen et al. [12] used servers to against inside offline keyword guessing attack. Wu et al. [13] presented a certificateless PEKS without public-key infrastructure. Zhang et al. [14] used a blockchain network to protect security of PEKS. Zhang et al. [15] gave a lattice-based scheme for industrial Internet of Things. Ma et al. [16] proposed a new framework of indistinguishability under a chosen-ciphertext-attack (IND-CCA) secure for PEKS. Miao et al. [17], Li et al. [18] and Cui et al. [19], respectively, proposed attribute-based encryption schemes with keyword search which are designed for multi-user with access controls.

Related Work. Attribute-based encryption with keyword search (ABEKS) [17,18,19] evolves from attribute-base encryption (ABE). ABE is a public-key encryption scheme which can realize that users with different key can decrypt the same cipher if and only if the users’ attributes meet the access structure. ABEKS inherits this property and adds a keyword search functionality. It is also designed for multi-user and with a strongly access control. However, the three schemes have an authority which is a fully trusted third party and there are bilinear pairings in their processes which need more assumption and more computing ability. Since a lot of schemes are proposed, none of them realize the functionality of multi-keyword search and multi-receiver with a keyword-based ranked result in the meanwhile. In particular, we design an interaction of participants which is different from previous ones in order to protect the privacy of trapdoor and result. Our proposed schemes realize the above-mentioned functionalities step by step and all are against the off-line keyword guess attack. Instead of widely-used pairing in PEKS, there are no more than modular exponentiation and modular multiplication in schemes which means lower security assumptions and potential computational efficiency.

Our Contribution. In this paper, we achieve the functionality of multiple keywords search, ranked result and multi-user. First we propose PEKS which can achieve searching multiple keyword at a time. It realizes a basic function with good efficiency and we can proved that this scheme can resist offline keyword guess attack. Then, an improved scheme with a ranked result is proposed. It balances the functionality with efficiency and it is proved that this scheme can resist offline keyword guess attack too. Furthermore, we introduce a private cloud in both schemes suited for the multi-user scene. Compared with other works, all of our schemes is based on traditional decisional Diffie–Hellman and big integer factorization assumption rather than a decisional bilinear Diffie–Hellman assumption. As we do not use any pairing operations, the scheme with modular exponentiation is considered to have more computing potential and more practicability.

**Figure 2:**
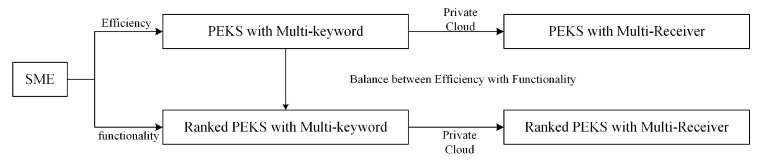
Relational graph of schemes.

Paper Outline. In this paper, we organize the details as follow. We introduce the model of PEKS in the Section 2 and security model in Section 3. The preliminary is described in the Section 4. A transformation of ElGamal encryption that we call Secure Match Encryption (SME) is showed here. Then, in the next section, a multi-keyword PEKS scheme is designed with a complex keyword structure. Although it has a fast computation efficiency, the search result is a precise match. In order to trade off the efficiency and functionality, a keyword-based ranked PEKS scheme is proposed in Section 6. The ranked PEKS can give a ranked result about the keyword, since it can return partial match result and give a record result about how many words is matched. In the Section 7, both PEKS are given in multi-receiver functionality via adding a private cloud. The analysis of performance is in the Section 8 (see Figure 2).

## 2. Model of PEKS

PEKS is used for users to obtain the right information from encrypted data without decryption. Different from the normal encryption, PEKS has a fixed flow which contains the sender(s), the cloud(s) and the user(s). The whole system is built for the user(s) to obtain the right information accurately and conveniently. That means the most of work is finished by the cloud(s), and the user(s) just spends a little of cost. In an IoT system managed by cloud servers, the sensors or other equipments take on the task of generating and sending data through simple processing. The cloud servers are data collector and manager which also have to serve their users. Realizing the different computing capacity of participants, the system is best left to the clouds to do more of the work. Users can get the most useful information at very little computational cost, and the process protects their privacy at all times. That is what PEKS is designed for, and that is how we design our solutions. We define the participants of the system in a specification description.

For a general scenarios, a PEKS system involves three participants including sender, server, receiver [3]. To extend extra functionality, an extra authority is drawn in the system. There is only one key pair of public and secret key in the system in which public key is published to all the participants and secret key is mastered by the Receiver. As an extension, a manager named Authority or Private cloud who take care of secret key is drawn into system and the Receiver as a group contains different users (receivers).

- Sender. It encrypts the data and generates the index of the data which contains a lot of keywords. For a public-key encryption, everyone even if the adversary can be a sender.
- Server Cloud. It receives and stores ciphertext from Sender, and execute the searching operation. Generally, Server is employed for most computation of the keyword searching and storage of encrypted data. It is supposed to be honest-but-curious, executing the protocol honestly but coveting the keywords from encrypted index curiously.
- Receiver. It generates the right trapdoor with the help of Authority and finishes the searching process with Server to obtain the right encrypted data. It is the owner of key pair, the true destination of delivered ciphertext and the one whom Server served for.
- Privacy Cloud. In a single user model, the most of computing tasks are undertaken which the user can choose to do by himself/herself. In a multi-user model, it manages the system secret key, verifies the legitimacy User, helps the verified User generate trapdoors and takes some computation of users.

The participants run algorithms according to a flow to realize a PEKS. For a more convenient description of the scheme, the algorithms is also formally defined before introducing a lot of mathematical formulas. We follow the previous results to describe the same algorithms. Public key encryption with keyword search (PEKS) scheme consists of the following four algorithms.

- **System Setup Algorithm**. Setup ($1^{k}$). It takes the security parameter $1^{k}$ as input, and outputs a pair of public key and secret key denoted as (pk,sk).
- **PEKS Algorithm**. PEKS ( pk,KW,data ). It takes the public key pk, a set of keywords KW and the data containing KW as input and outputs a ciphertext with a searchable index $\left(C,Index_{KW}\right)$.
- **Trapdoor Generation Algorithm**. Trapdoor ($pk,sk,KW^{\prime }$ ). It takes the public key pk, the secret key and a set of keywords $KW^{\prime }$ as input and outputs a trapdoor with $KW^{\prime }$ denoted $T_{KW^{\prime }}$.
- **Test Algorithm**. Test ($pk,Index_{KW},T_{KW^{\prime }}$). It takes the public key pk, a searchable index $Index_{KW}$ and a trapdoor with $T_{KW^{\prime }}$ as input. If $KW=KW^{\prime }$, it outputs 1 or if not it output 0.

## 3. Security Model

The security model is designed to prove secure under an adversary’s attack. The provable security of a scheme is considered as an important basis of public-key cryptography. Solutions that cannot be proven by a provable security system generally have undetected attacks. In order to eliminate risk, we use the semantic-security of encryption to define the security of PEKS. It is used to prove that PEKS will not reveal any information about the keyword KW.

Keyword guessing attack (KGA) is a common tool used by an adversary to break down schemes of PEKS. Because anyone can finish the encryption and build an index for a certain keyword. Given a trapdoor, he or she can detect if the trapdoor is about a certain keyword or not. To resist KGA, there are two means considered to take effect. First, restrict the range of people who can build the index. Second, the result should be obtained by limited people. Whether cutting off the process of encryption and index building or imposing restrictions on the result-unfold people is to stop the chance to irrelevant people guessing the keyword. For a strict definition, we follow the definition and model of anti-KGA secure which was imposed by [4].

- Initalization: System runs the key generation algorithm and publishes the pk and other public parameters. Allow the adversary to choose any two keywords in the range of keywords, marked by $k_{1},k_{2}$.
- Phase1: The adversary can query trapdoor of any keyword in the range of keywords. It obtains the right results. This process can be repeated many times in polynomial time.
- Challenge: Uniformly choose *b*, so that the probability of b=1 or b=2 is either 0.5. Generate the trapdoor of $k_{b}$, and return it to the adversary.
- Phase2: Repeat as the second phase.
- Test: The adversary outputs a guess of *b* denoted as $b^{\prime }$.

The advantage of adversary denoted ${Adv}_{A}$ is defined by

$${Adv}_{A}=P\left(b=b^{\prime }\right)-\frac{1}{2}=\frac{1}{2}\left|1-2P\left(b=b^{\prime }\right)\right|.$$

If the advantage is negligible, the scheme is against KGA.

## 4. Preliminary

In this section, we introduce the mathematical basis of our schemes. All the designs in this article are based on finite field assumptions which are regarded as nondeterministic-polynomial-time algebra problems. If the assumption is held, our schemes can be proved secure in the standard model.

Decisional Diffie–Hellman assumption (DDH). (G,·) is a multiplicative cyclic group with order *n*, and *g* is an element of *G*. Given $g^{a},g^{b}$ and $g^{c}$, it is difficult to judge whether c=ab (mod *n*) or not without a,b and *c*.

It has been proved that if the decisional Diffie–Hellman assumption is held, ElGamal Encryption is of semantic security [20].

Prime factorization assumption (PF). Given n=pq, in which *p* and *q* both are big primes and |p|≈|q|, it is difficult to compute *p* and *q*.

It has been proved that if the Prime factorization assumption is held, RSA Encryption is of semantic security [21].

In order to structure our scheme, we put forward a variant scheme of ElGmal Encryption which we record as Secure Match Encryption (SME).

Key Generation: with security parameter λ as input, the algorithms select public parameters (g,p,q). *p* and *q* are big primes and *g* is the generator of subgroup of $Z_{p}$ with order *q*. Choose variable *x* uniformly at random from $Z_{q}$ as private key and compute $y=g^{x}$ mod *p* as public key.

Encryption: with public parameters (g,p,q), public key *y* and plaintext 1<m<q as input, this algorithm chooses variable *r* uniformly at random from $Z_{q}$ and computes $c_{1}=g^{r}$ mod *p*, $c_{2}=m\cdot y^{r}$ mod *p*, and $c_{3}=g^{m}y^{r}$ mod *p*. The result is ciphertext $c=(c_{1},c_{2},c_{3})$.

Decryption: with public parameters (g,p,q), private key *x* and ciphertext *c*, the algorithm computes $m^{\prime }=\left(c_{1}/c_{2}^{x}\right)$ mod *p*.

Trapdoor Generation: with public parameters (g,p,q), public key *y* and keyword $1<m^{\prime }<q$, as input, this algorithm chooses variable *k* uniformly at random from $Z_{q}$ and computes $c_{1}^{\prime }=g^{k}$ and $c_{3}^{\prime }=g^{-m^{\prime }}y^{k}$,

Test: compute $c_{s}=\left(c_{s1}=c_{1}\cdot c_{1}^{\prime }=g^{r+k},c_{s3}=c_{3}\cdot c_{3}^{\prime }=g^{m-m^{\prime }}y^{r+k}\right)$$c_{s1}^{x}=c_{s3}$ or not.

SME is a transformation of ElGamal. Using the SME scheme, we can easily get a PEKS scheme for a single keyword search. Different from traditional PEKS, the result of the Test is revealed to the user with the secure key *x*, and most computation of test can be finished by any distrustful third part or public cloud. We design this mechanism aimed at privacy protection.

## 5. PEKS without Bilinear Pairings

In a cloud-based IoT system, there are kinds of equipment that have different capabilities. Particularly, some sensors have little computing power and the cloud in the middle of the system has much computing power. The PEKS with bilinear pairings is not suitable for IoT systems because bilinear pairings are not friendly to lightweight devices. Pairing-based cryptography is firstly imposed by Koblitz, et al. [22]. Pairings are used to construct cryptographic systems, such as identity-based encryption and attribute-based encryption schemes. However, the cost of computing pairings is more than computing modular exponentiation or modular multiplication. Therefore, the schemes without pairings are needed by lightweight devices. Then the PEKS with multi-keyword search at once is designed which uses a complex structure with homomorphic calculation instead of pairings.

### 5.1. Scheme

There are five algorithms as follow.

- **Setup($1^{k}$)**. With a security parameter *k*, the system chooses two big prime *p* and *q* where p−1 and q−1 both have big prime factors. Compute n=p·q and Φ(n)=(p−1)(q−1) which is called euler function. Uniformly choose a random number *e* such that 1<e<ϕ(n) and gcd(e,ϕ(n))=1 where gcd means greatest common divisor function. Compute *d* such that d·e=1mod(ϕ(n)) with Extended Euclidean algorithm. Choose an element *g* with order ϕ(n) in $Z_{n}^{*}$ where $Z_{n}^{*}$ is the multiplicative group in integers module *n* without 0 and compute $y=g^{e}$. Then output y,g,n as the public key pk and e,d,p,q as the secret key sk. Randomly choose a number *l* such that l<p, l<q and |l|=min(|p|,|q|). There exists a usable Hash function.
- **PEKS(pk,KW)**. For $keyword_{i}\in KW$, compute $kw_{i}=Hash\left(keyword_{i}\right)$ and uniformly choose a random number $r_{i}$ such that $r_{i}\in \left[1,l\right]$. Compute and output $\left(c_{i1},c_{i2}\right)=\left(g^{r_{i}},y^{r_{i}}g^{kw_{i}}\right)$ as PEKS.
- **Trapdoor($\mathrm{pk},\mathrm{sk},{\mathrm{KW}}^{\prime }$)**. Choose a random number *x* such that x∈[1,l]. For $keyword_{i}\in KW^{\prime }$, compute $kw_{i}^{\prime }=Hash\left(keyword_{i}\right)$ and choose a random number $r_{i}^{\prime }$ such that $r_{i}^{\prime }\in \left[1,l\right]$. Compute and output
  $\left(t_{1},t_{2}\right)=\left(\prod_{kw_{i}^{\prime }\in KW^{\prime }}{\left(g^{r_{i}^{\prime }}\right)}^{x^{i}},\prod_{kw_{i}^{\prime }\in KW^{\prime }}{\left(y^{r_{i}^{\prime }}g^{-kw_{i}^{\prime }}\right)}^{x^{i}}\right)=\left(g^{\sum_{kw_{i}^{\prime }\in KW^{\prime }}r_{i}^{\prime }x^{i}},g^{\sum_{kw_{i}^{\prime }\in KW^{\prime }}r_{i}^{\prime }ex^{i}}g^{\sum_{kw_{i}^{\prime }\in KW^{\prime }}-kw_{i}^{\prime }x^{i}}\right)$ with *x* as $T_{KW^{\prime }}$.
- **Search($\mathrm{pk},x,T_{{\mathrm{KW}}^{\prime }},\mathrm{PEKS}$)**. For any message’s PEKS, compute and output
  $$\begin{array}{l} \;\;\;\;\;(R_{1},R_{2})\\ =(\prod_{kw_{i}\in KW}{\left(g^{r_{i}}\right)}^{x^{i}}\cdot t_{1},\prod_{kw_{i}\in KW}{\left(g^{r_{i}e}g^{kw_{i}}\right)}^{x^{i}}\cdot t_{2})\\ =(g^{\sum_{kw_{i}\in KW}r_{i}x^{i}}\cdot t_{1},g^{\sum_{kw_{i}\in KW}r_{i}ex^{i}}g^{\sum_{kw_{i}\in KW}kw_{i}x^{i}}\cdot t_{2}) \end{array}$$
  as ResEnc.
- **Test (pk,ResEnc,sk)**. With *d*, judge $R_{1}\overset{?}{=}{R_{2}}^{d}$. If the equation holds, output 1, otherwise outputs 0. With p,q,e, judge $R_{2}\overset{?}{=}{R_{1}}^{e}$. If the equation holds, output 1, otherwise outputs 0.

Correctness. Here is the proof that our construction meets the requirements of correct definition as claimed above.

If $KW^{\prime }$ matches PEKS ’s KW, the proof is as follow.

$$\begin{array}{l} \;\;\;\;\;(R_{1},R_{2})\\ =(\prod_{kw_{i}\in KW}{\left(g^{r_{i}}\right)}^{x^{i}}\cdot t_{1},\prod_{kw_{i}\in KW}{\left(g^{r_{i}e}g^{kw_{i}}\right)}^{x^{i}}\cdot t_{2})\\ =(g^{\sum_{kw_{i}\in KW}r_{i}x^{i}}\cdot t_{1},g^{\sum_{kw_{i}\in KW}r_{i}ex^{i}}g^{\sum_{kw_{i}\in KW}kw_{i}x^{i}}\cdot t_{2})\\ =(g^{\sum_{kw_{i}\in KW}r_{i}x^{i}}\cdot g^{\sum_{kw_{i}^{\prime }\in KW^{\prime }}r_{i}^{\prime }x^{i}},g^{\sum_{kw_{i}\in KW}r_{i}ex^{i}}g^{\sum_{kw_{i}\in KW}kw_{i}x^{i}}\cdot g^{\sum_{kw_{i}^{\prime }\in KW^{\prime }}r_{i}^{\prime }ex^{i}}g^{\sum_{kw_{i}^{\prime }\in KW^{\prime }}-kw_{i}^{\prime }x^{i}})\\ =(g^{\sum_{kw_{i}\in KW}r_{i}x^{i}+\sum_{kw_{i}^{\prime }\in KW^{\prime }}r_{i}^{\prime }x^{i}},g^{\sum_{kw_{i}\in KW}r_{i}ex^{i}+\sum_{kw_{i}^{\prime }\in KW^{\prime }}r_{i}^{\prime }ex^{i}}g^{\sum_{kw_{i}\in KW}kw_{i}x^{i}+\sum_{kw_{i}^{\prime }\in KW^{\prime }}-kw_{i}^{\prime }x^{i}})\\ =(g^{\sum_{kw_{i}\in KW}\left(r_{i}+r_{i}^{\prime }\right)x^{i}},g^{\sum_{kw_{i}\in KW}\left(r_{i}+r_{i}^{\prime }\right)ex^{i}}g^{\sum_{kw_{i}\in KW}\left(kw_{i}-kw_{i}\right)x^{i}})\\ =\left(R_{1},{R_{1}}^{e}\right)=({R_{2}}^{d},R_{2}). \end{array}$$

### 5.2. Security Proof

**Theorem** **1.** *The proposed PEKS scheme is semantically secure against offline KGA in the random oracle model if PF and DDH assumption are both hard to solve in probabilistic polynomial time (PPT).*

**Proof.**  First, we formalize the assumptions in algebra form.

If the PF assumption holds, RSA is security. With a security parameter *k*, given n=pq in which *p* and *q* are both big prime, it is difficult for a probabilistic polynomial time adversary to calculate *p* and *q*. Choose a random number *e* such that 1<e<ϕ(n)=(p−1)(q−1), it is difficult to obtain *d* such that edmodϕ(n)=1 without *p* and *q*.

If the DDH assumption holds, ElGamal Encryption is security. With a security parameter of *k*, a group $G=Z_{n}^{*}$, and a element *g* of *G* with the order ϕ(n), make e:1<e<ϕ(n) as ElGamal Encryption secret key and $y=g^{e}$ as public key. Arbitrarily choose two message $m_{1},m_{2}$, and toss a coin to decide *b*. Randomly choose a number $r^{*}$ such that $1<r^{*}<\phi \left(n\right)$, and compute $g^{r^{*}},y^{r^{*}}g^{m_{b}}$. It is difficult for a probabilistic polynomial time adversary to judge *b* without *e*.

Suppose there was a PPT adversary who can break up our scheme in the game defined above. In order to train the adversary’s ability, the simulator uses the security model to simulate the real protocol.

- Initialization. The simulator runs the Setup algorithm, and outputs the $pk=g^{e},g,n$. The simulator uniformly randomly chooses a number with parameter *k* as *l*.
- Phase1. In this phase, the Adversary can query the trapdoor of any keyword set and the search result of any trapdoor.
  If the keyword set that Adversary queries is $KW_{j}$, in order to answer $Query(Trapdoor\left(pk,sk,KW^{\prime }\right))$, Simulator chooses a random number *x* such that x∈[1,l], and a random number $r_{i}^{\prime }$ such that $r_{i}^{\prime }\in \left[1,l\right]$ for every keywords $kw_{i}^{\prime }\in KW_{j}$. Simulator computes and outputs $\left(t_{1},t_{2}\right)=\left(g^{\sum_{kw_{i}^{\prime }\in KW_{j}}r_{i}^{\prime }x^{i}},y^{\sum_{kw_{i}^{\prime }\in KW_{j}}r_{i}^{\prime }x^{i}}g^{\sum_{kw_{i}^{\prime }\in KW^{\prime }}-kw_{i}^{\prime }x^{i}}\right)$ with *x* as $T_{KW_{j}}$.
  In order to answer $Query(Search\left(pk,x,T_{KW_{j}},PEKS\right)$, the Simulator computes and outputs
  $$\begin{array}{l} \left(R_{1},R_{2}\right)=\left(\prod_{kw_{i}\in KW}{\left(g^{r_{i}}\right)}^{x^{i}}\cdot t_{1},\prod_{kw_{i}\in KW}{\left(g^{r_{i}e}g^{kw_{i}}\right)}^{x^{i}}\cdot t_{2}\right)\\ =(g^{\sum_{kw_{i}\in KW}r_{i}x^{i}}\cdot t_{1},g^{\sum_{kw_{i}\in KW}r_{i}ex^{i}}g^{\sum_{kw_{i}\in KW}kw_{i}x^{i}}\cdot t_{2}) \end{array}$$
  as ResEnc.
- Challenge. Simulator asks the adversary to choose two keyword sets $KW_{0}$ and $KW_{1}$. Simulator tosses a coin to get $b^{\prime }$ which is 0 or 1. Simulator randomly chooses numbers $r^{**},x^{**}$ such that $1<r^{**}<\phi \left(n\right),1<x^{**}<\phi \left(n\right)$, computes and outputs $\left(g^{r^{**}},y^{r^{**}}g^{-m_{b^{\prime }}}\right)$ with $x^{**}$ as $T_{KW_{b^{\prime }}}$. Simulator computesand outputs $\left(g^{r^{*}}\cdot g^{r^{**}},y^{r^{*}}g^{m_{b}}\cdot y^{r^{**}}g^{-m_{b^{\prime }}}\right)$ as ResEnc.
- Phase2. Repeat Phase 1 except queries of $KW_{0}$ and $KW_{1}$’s trapdoor and the search result of $T_{KW_{b^{\prime }}}$.
- Test. Let the adversary output a guess $b^{\prime \prime }$ of $b^{\prime }$ and the simulator uses $b^{\prime \prime }$ as the guess of *b*.

The advantage of Adversary is

$Adv_{A}$ = $\frac{1}{2}-Prob(b^{\prime \prime }$ = $b^{\prime })$ = $\frac{1}{2}-Prob(b^{\prime \prime }$ = b) = $Adv_{S}$.

Because the ElGamal Encryption is secure, the advantage of Simulator is negligible. So our scheme is proved secure against the Off-Line KGA. □

### 5.3. Implementation

In this section, we elaborate details of PEKS. As described above in Section 3, there are Sender, Receiver, Public cloud and an optional private cloud which need more security requirement. At the beginning of the system, there is a keyword list that contains all the keywords for our system’s need. The keywords are fixed order by dictionary sequence or any other fixed sequence. As a result, the system needs every keyword can be obtained with an order number *i*. If the amount of keyword set is *N*, we record the keywords with $kw_{i}$, in which i∈[1,N]. As it is stored in form of plaintext, any participant can obtain the list, keywords and their order numbers. The Receiver runs the Setup algorithm, publishes the public parameter and public key and stores the secret key himself/herself. For reduced representation, there are default ordinary encryption schemes with semantic security which are used to keep the content of the message confidential and transfer the message. We will not mention it again in the article.

**Figure 3:**
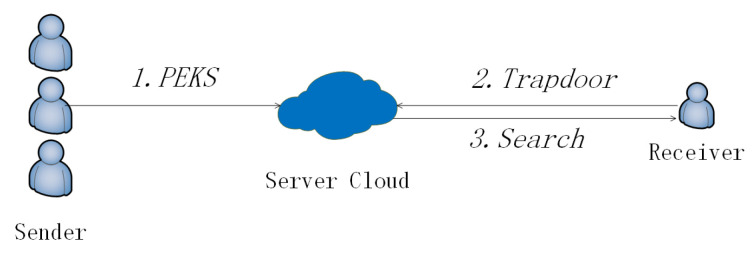
Public-key encryption with keyword search (PEKS) without a Private Cloud.

**Figure 4:**
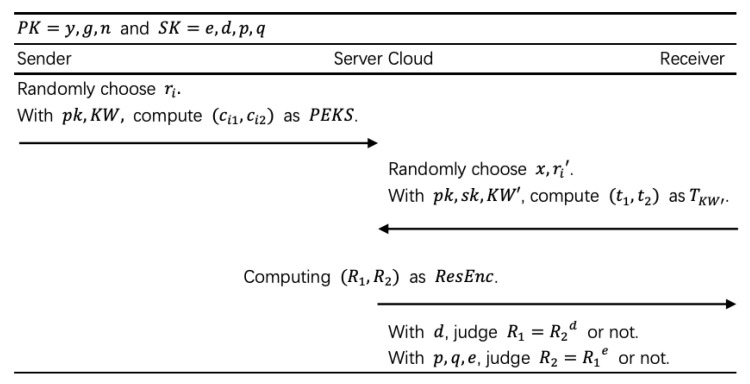
Flow of PEKS.

For a scene without a private cloud (Figure 3), the flow is as follow (Figure 4). When any Sender wants to transfer a message *m* to a Receiver, he/she runs a keyword extraction algorithm that generates keywords KW for the message. Then he/she runs the PEKS algorithm with input pk,KW and outputs PEKS. The Sender transfers PEKS with an ordinary encryption Enc(m) to the Public Cloud. When a Receiver wants to search keywords $KW^{\prime }$, he/she runs Trapdoor algorithm with input $pk,sk,KW^{\prime }$ and outputs $T_{KW^{\prime }}$ and *p*. He/she sends the $T_{KW^{\prime }}$ and *p* to the Public Cloud. The Public Cloud is responsible for receiving and storing PEKS. When the Public Cloud receives the search query, it runs the Search algorithm with input $pk,T_{KW^{\prime }},p,PEKS$ and outputs encrypted results. Absolutely, the Public Cloud has the most of the computation task in our scheme. The Public Cloud sends the encrypted result to the Receiver. At last, the Receiver uses his/her sk and ResEnc to run the Test algorithm. The result of the search can only be obtained by the sk’ owner.

**Figure 5:**
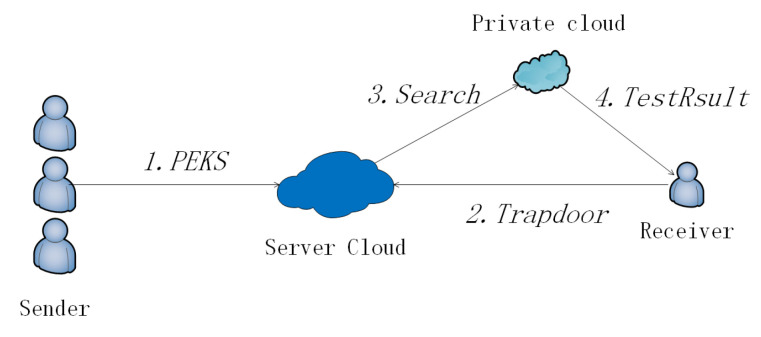
PEKS with the Private Cloud.

For a scene with a private cloud (see Figure 5), the flow is as follow. When any Sender wants to transfer a message *m* to a Receiver, he/she runs a keyword extraction algorithm that generates keywords KW for the message. Then he/she runs the PEKS algorithm with input pk,KW and outputs PEKS. The Sender transfers PEKS with an ordinary encryption Enc(m) to the Public Cloud. When Receiver wants to search keywords $KW^{\prime }$, he/she runs Trapdoor algorithm with input $pk,sk,KW^{\prime }$ and outputs $T_{KW^{\prime }}$ and *p*. He/she sends the $T_{KW^{\prime }}$ and *p* to the Public Cloud. Public Cloud is responsible for receiving and storing PEKS. When Public Cloud receives the search demand, it runs the Search algorithm with input $pk,T_{KW^{\prime }},p,PEKS$ and outputs encrypted results. Absolutely, Public Cloud has the most of the computation task in our scheme. Public Cloud sends the encrypted result to the Private Cloud. At last, the Cloud uses his/her sk and ResEnc to run the Test algorithm. The result will be returned to the receiver.

### 5.4. Privacy Analysis

We prove our PEKS scheme secure against keyword guessing attack. That means no information of keywords can be leaking. To realize this function, we use salt number *x* to protect kw. A different keyword is covered by *x*’s exponent of a different order. For finite field, the DDH and PF hold, the value of kw is secure, that means the keyword is protected very well.

For the IoT system, Sensors or other devices send PEKS with data to the cloud. The cloud obtains no useful information about the real data. Cloud provides service for users without knowing what the users get from cloud. If choosing a private cloud, the nearly whole computing is done by the cloud. This is friendly to lightweight devices which is common in the IoT system.

## 6. Keyword-Based Ranked Search

To achieve keyword-based sorts, we use the technology of order-preserving encryption. When the protocol is executing, users use *p* to generate the trapdoor of some keywords. As well, *p* can achieve order-preserving for the keywords. In this section, we give a detailed description of our schemes.

At the beginning of the system, there is a keyword list that contains all the keywords for our system’s needs. The keywords are fixed order by dictionary sequence or any other fixed sequence. As a result, system needs every keyword can be obtained with an order number *i*. If the amount of keyword set is *N*, we record the keywords with $kw_{i}$, in which i∈[1,N]. As it is unecrypted, any participant can obtain the list, keywords and their order numbers.

### 6.1. Scheme

- **Setup($1^{k}$)**. With a security parameter *k*, the system chooses two big prime p,q where p−1 and q−1 both have big prime factors. Compute n=p·q and Φ(n)=(p−1)(q−1) which is called euler function. Uniformly choose a random number *e* such that 1<e<ϕ(n) and gcd(e,ϕ(n))=1 where gcd means the greatest common divisor function. Compute *d* such that d·e=1mod(ϕ(n)) with an Extended Euclidean algorithm. Choose an element *g* with order ϕ(n) in $Z_{n}^{*}$ and compute $y=g^{e}$. Then output y,g,n as the public key and p,q,e,d as the secret key. There exists a usable Hash function.
- **PEKS(pk,KW)**. For $keyword_{i}\in KW$, compute $kw_{i}=Hash\left(keyword_{i}\right)$ and uniformly choose a random number $r_{i}$ such that $r_{i}\in \left[1,n\right]$. Compute and output $\left(c_{i1},c_{i2}\right)=\left(g^{r_{i}},y^{r_{i}}g^{kw_{i}}\right)$ as PEKS. For $keyword_{i}\notin KW$, compute $kw_{i}=Hash\left(keyword_{i}\right)$ and uniformly choose a random number $r_{i}$ such that $r_{i}\in \left[1,n\right]$. Compute and output $\left(c_{i1},c_{i2}\right)=\left(g^{r_{i}},y^{r_{i}}g^{kw_{i}-1}\right)$ as PEKS.
- **Trapdoor($\mathrm{pk},\mathrm{sk},{\mathrm{KW}}^{\prime }$)**. For $keyword_{i}\in KW^{\prime }$, compute $kw_{i}=Hash\left(keyword_{i}\right)$ and choose a random number $x_{i}$ such that $r_{i}^{\prime },x_{i}\in \left[1,n\right]$ and $x_{i}\;mod\;\left(p-1\right)\neq 0$. For $keyword_{i}\notin KW^{\prime }$, compute $kw_{i}=Hash\left(keyword_{i}\right)$, choose a random number $r_{i}^{\prime },a_{i},b_{i}$ such that $a_{i},b_{i}\in \left[1,n\right]$ and $a_{i},b_{i}\;mod\;\left(q-1\right)\neq 0$, and compute $x_{i}=b_{i}{\left(p-1\right)}^{a_{i}}$modϕ(n). Compute and output
  $$\begin{array}{l} \left(t_{1},t_{2}\right)={\left(\prod_{i\in [1,N]}(g^{r_{i}^{\prime }}\right)}^{x_{i}},\prod_{kw_{i}\in KW^{\prime }}{\left(y^{r_{i}^{\prime }}g^{-kw_{i}}\right)}^{x_{i}}\prod_{kw_{i}\notin KW^{\prime }}{\left(y^{r_{i}^{\prime }}g^{1-kw_{i}}\right)}^{x_{i}})\\ =(g^{\sum_{i\in [1,N]}r_{i}^{\prime }x_{i}},g^{\sum_{i\in [1,N]}r_{i}^{\prime }ex_{i}}g^{\sum_{kw_{i}\in KW}-kw_{i}x_{i}+\sum_{kw_{i}\notin KW}\left(1-kw_{i}\right)x_{i}}) \end{array}$$
  with $x_{i}$ as $T_{KW^{\prime }}$.
- **Search($\mathrm{pk},x,\mathrm{PEKS},T_{{\mathrm{KW}}^{\prime }}$)**. For $keyword_{i}\in KW^{\prime }$, compute $kw_{i}=Hash\left(keyword_{i}\right)$. Compute and output
  $$\left(R_{1},R_{2}\right)=\left(\prod {\left(c_{i1}\right)}^{x_{i}}\cdot t_{1},\prod {\left(c_{i2}\right)}^{x_{i}}\cdot t_{2}\right)=\left(g^{\sum r_{i}x_{i}}\cdot t_{1},g^{\sum r_{i}ex_{i}}g^{\sum_{kw_{i}\in KW}kw_{i}x_{i}+\sum_{kw_{i}\notin KW}\left(kw_{i}-1\right)x_{i}}\cdot t_{2}\right)$$
  as ResEnc.
- **Test(pk,ResEnc,x,sk)**. With *e* and *q*, compute $R=\frac{{R_{2}}^{q-1}}{{\left({R_{1}}^{q-1}\right)}^{e}}$. With *d*, it can compute ${R_{2}}^{d}$ and judge whether ${R_{2}}^{d}\overset{?}{=}R_{1}$ or not.

Correctness. Here is the proof that our construction meets the requirements of the correct definition as claimed above.

$$R=\frac{{R_{2}}^{q-1}}{{\left({R_{1}}^{q-1}\right)}^{e}}=\frac{{\left(\prod {\left(c_{i2}\right)}^{x_{i}}\cdot t_{2}\right)}^{q-1}}{{\left(\prod {\left(c_{i1}\right)}^{x_{i}}\cdot t_{1}\right)}^{(q-1)e}}.$$

Because $x_{i}=b_{i}{\left(p-1\right)}^{a_{i}}$ for every keywords $kw_{i}^{\prime }\notin KW^{\prime }$, $\left(x_{i}\right)\left(q-1\right)\;mod\;\phi \left(n\right)=0$. Then

$$\begin{array}{ll} R & =(\prod_{kw_{i}\in KW^{\prime }\cap KW}g^{e\left(r_{i}+r_{i}^{\prime }\right)x_{i}\left(q-1\right)}g^{\left(kw-kw\right)x_{i}\left(q-1\right)}\;\;\;\;\cdot \prod_{kw_{i}\in KW^{\prime },kw_{i}\notin KW}g^{e\left(r_{i}+r_{i}^{\prime }\right)x_{i}\left(q-1\right)}g^{\left(kw-kw+1\right)x_{i}\left(q-1\right)})\\ & \;\;\;\;/\prod_{kw_{i}\in KW^{\prime }}g^{e\left(r_{i}+r_{i}^{\prime }\right)x_{i}\left(q-1\right)}\\ & =\prod_{kw_{i}\in KW^{\prime },kw_{i}\notin KW}g^{x_{i}\left(q-1\right)} \end{array}$$

If $KW^{\prime }=KW$, R=1. In general, the keywords’ amount of once search is less than 10. So it is easy to screen the match keywords from others using *R*.

### 6.2. Security Proof

**Theorem** **2.** *The proposed PEKS scheme is semantically secure against offline KGA in the random oracle model if ElGmal and RSA both are hard to solve in probabilistic polynomial time.*

**Proof.**  First, we formalize the assumptions in algebra form.

If the PF assumption holds, RSA is security. With a security parameter *k*, given n=pq in which *p* and *q* are both big prime, it is difficult for a probabilistic polynomial time adversary to calculate *p* and *q*. Choose a random number *e* such that 1<e<ϕ(n)=(p−1)(q−1), it is difficult to obtain *d* such that edmodϕ(n)=1 without *p* and *q*.

If the DDH assumption holds, ElGamal Encryption is security. With a security parameter *k*, a group $G=Z_{n}^{*}$, and a element *g* of *G* with the order ϕ(n), make e:1<e<ϕ(n) as ElGamal Encryption secret key and $y=g^{e}$ as public key. Arbitrarily choose two message $m_{1},m_{2}$, and toss a coin to decide *b*. Randomly choose a number $r^{*}$ such that $1<r^{*}<\phi \left(n\right)$, and compute $g^{r^{*}},y^{r^{*}}g^{m_{b}}$. It is difficult for a probabilistic polynomial time adversary to judge *b* without *e*.

Suppose there was a PPT adversary who can break up our scheme in the game defined above. In order to train the adversary’s ability, the simulator uses the security model to simulate the real protocol.

- Initialization. The simulator runs the Setup algorithm, and outputs the $pk=g^{e},g,n$. Simulator uniformly randomly chooses a number with parameter *k* as *l*.
- Phase1. In this phase, the Adversary can query the trapdoor of any keyword set and the search result of any trapdoor.
  If the keyword set that Adversary queries is $KW_{j}$, in order to answer $Query(Trapdoor\left(pk,sk,KW^{\prime }\right))$, Simulator chooses a random number *x* such that x∈[1,l], and chooses a random number $r_{i}^{\prime }$ such that $r_{i}^{\prime }\in \left[1,l\right]$ for every keywords $kw_{i}^{\prime }\in KW_{j}$. Simulator computes and outputs
  $$\begin{array}{l} \left(t_{1},t_{2}\right)={\left(\prod_{i\in [1,N]}(g^{r_{i}^{\prime }}\right)}^{x_{i}},\prod_{kw_{i}\in KW^{\prime }}{\left(y^{r_{i}^{\prime }}g^{-kw_{i}}\right)}^{x_{i}}\prod_{kw_{i}\notin KW^{\prime }}{\left(y^{r_{i}^{\prime }}g^{1-kw_{i}}\right)}^{x_{i}})\\ =(g^{\sum_{i\in [1,N]}r_{i}^{\prime }x_{i}},g^{\sum_{i\in [1,N]}r_{i}^{\prime }epx_{i}}g^{\sum_{kw_{i}\in KW}-kw_{i}x_{i}+\sum_{kw_{i}\notin KW}\left(1-kw_{i}\right)x_{i}}). \end{array}$$
  In order to answer $Query(Search\left(pk,x,T_{KW_{j}},PEKS\right)$, Simulator computes and outputs
  $$\begin{array}{ll} \left(R_{1},R_{2}\right) & =\left(\prod {\left(c_{i1}\right)}^{x_{i}}\cdot t_{1},\prod {\left(c_{i2}\right)}^{x_{i}}\cdot t_{2}\right)\\ =(g^{\sum r_{i}x_{i}},g^{\sum r_{i}ex_{i}}g^{\sum_{kw_{i}\in KW}kw_{i}x_{i}+\sum_{kw_{i}\notin KW}\left(kw_{i}-1\right)x_{i}}) \end{array}$$
  as ResEnc.
- Challenge. The Simulator asks the adversary to choose two keyword sets $KW_{0}$ and $KW_{1}$. The Simulator tosses a coin to get $b^{\prime }$ which is 0 or 1. The Simulator randomly chooses numbers $r^{**},x^{**}$ such that $1<r^{**}<\phi \left(n\right),1<x^{**}<\phi \left(n\right)$, computes and outputs $\left(g^{r^{**}},y^{r^{**}}g^{-m_{b^{\prime }}}\right)$ with $x^{**}$ as $T_{KW_{b^{\prime }}}$. The Simulator computes and outputs $\left(g^{r^{*}}\cdot g^{r^{**}},y^{r^{*}}g^{m_{b}}\cdot y^{r^{**}}g^{-m_{b^{\prime }}}\right)$ as ResEnc.
- Phase2. Repeat Phase 1 except queries of $KW_{0}$ and $KW_{1}$’s trapdoor and the search result of $T_{KW_{b^{\prime }}}$.
- Test. Let the adversary outputs a guess $b^{\prime \prime }$ of $b^{\prime }$ and the simulator uses $b^{\prime \prime }$ as the guess of *b*.

The advantage of Adversary is

$$Adv_{A}=\frac{1}{2}-Prob\left(b^{\prime \prime }=b^{\prime }\right)=\frac{1}{2}-Prob\left(b^{\prime \prime }=b\right)=Adv_{S}$$.

Because the ElGamal Encryption is secure, the advantage of Simulator is negligible. So our scheme is proved secure against the Off-Line KGA. □

### 6.3. Implementation of Ranked Scheme

The keyword-based ranked scheme is similar to the previous exact match one. It provides optional flows with a private cloud or without a private cloud which can decide the computation of the Receiver. The order-fixed keywords need to be preprocessed before the system setup. We just elaborate details of difference.

A private cloud with a secret key *d* can judge whether a trapdoor precisely matches PEKS. To realize the keyword-based ranked functionality, the Receiver must compute open the ResEnc with his/her secret key q,e. In fact, the previous scheme can publish *p* and *q* so that everyone can generate trapdoors of any keyword set. It does not leak any privacy of trapdoors but leads to that the secret key *e* and *d* can easily compute by each other. In the keyword-based ranked scheme, only Receiver holds p,q and is able to generate trapdoors. *p* and *q* are more associated with security. As described above, the amount of keywords generally is under 10. So it is acceptable to compute the rank for a PEKS.

### 6.4. Privacy Analysis

We prove our Ranked PEKS scheme secure against keyword guessing attack. As the same as PEKS, Ranked PEKS is friendly to IoT devices and the privacy of data is protected very well. To realized ranked functionality, we let every kw in the list participate in the operation. This design leads to consuming more computing but obtaining ranked results. However, as we designed, the most of operation is done by cloud. The IoT devices and users do not need more computing power.

## 7. PEKS for Multi-User

A single-user system is enough for personal use, but data sharing with family or friends can bring more fun. In the field of industry, collaboration among colleagues is inseparable from information sharing. Industrial Internet of Things (IIoT) is particularly popular in improving productivity. It is imperative to design lightweight systems for multiple users. In this section, we transfer our schemes to multi-receiver PEKS. The algorithm is partially changed and most computation is finished by the public cloud and private cloud.

### 7.1. Multi-User PEKS

**Figure 6:**
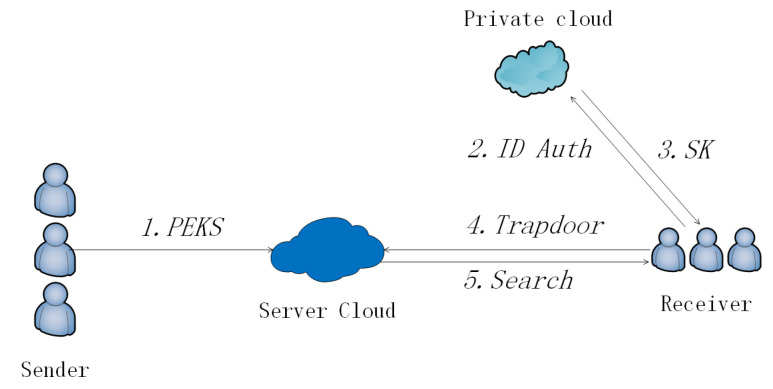
Multi-user PEKS (MU-PEKS).

**Figure 7:**
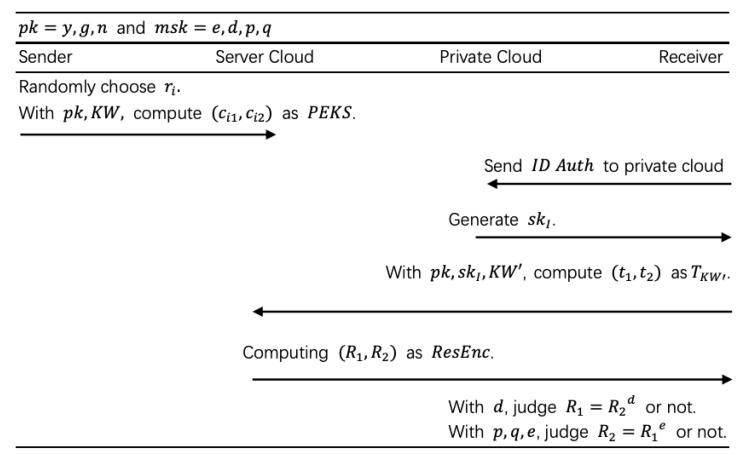
Flow of MU-PEKS.

In this subsection, we use our schemes to construct a multi-user PEKS (Figure 6) and Figure 7 shows the detailed flow.

- **MU-PEKS.Setup ($1^{k}$)**. Run $PEKS.Setup(1^{k})$ and output y,g,n as public key pk and p,q,e,d as master secret key msk.
- **MU-PEKS.PEKS (pk,KW)**. Run PEKS.PEKS(pk,KW) and output the result as PEKS.
- **Mu-PEKS.Secretkey ($\mathrm{pk},\mathrm{msk},{\mathrm{IDAuth}}_{I}$)**. The input of this algorithm is the system’s pk, msk and IDAuth, where IDAuth is an authority of identity. It outputs the secret key $sk_{I}$ for $Receiver_{I}$. Uniformly choose a random number $e_{I}$ such that $1<e_{I}<\phi \left(n\right)$ and $gcd(e_{I},\phi \left(n\right))=1$. Compute $d_{I}$ such that $d_{I}\cdot e_{I}=1\;mod\;\phi \left(n\right)$ with Extended Euclidean algorithm. Output $d,e_{I}$ as $Receiver_{I}$’s $sk_{I}$ and $d_{I}$ as $Receiver_{I}$’s $pk_{I}$.
- **MU-PEKS.Trapdoor ($\mathrm{pk},\mathrm{msk},{\mathrm{KW}}^{\prime },{\mathrm{pk}}_{I}$)**. Run
  $PEKS.Trapdoor(pk,msk,KW^{\prime })$ and output $\left(t_{1},t_{2}\right)=\left(PEKS.t_{1},{PEKS.t_{2}}^{d_{I}}\right)$ with *x* as $T_{KW^{\prime }}$.
- **MU-PEKS.Search ($\mathrm{pk},x,{\mathrm{KW}}^{\prime },{\mathrm{pk}}_{I}$)**. For any message’s PEKS, compute and output $\left(R_{1},R_{2}\right)=\left(\prod_{kw_{i}\in KW}{\left(g^{r_{i}}\right)}^{x^{i}}\cdot t_{1},{\left(\prod_{kw_{i}\in KW}{\left(g^{r_{i}e}g^{kw_{i}}\right)}^{x^{i}}\right)}^{d_{I}}\cdot t_{2}\right)$ as ResEnc.
- **Mu-PEKS.Test ($\mathrm{pk},\mathrm{ResEnc},{\mathrm{sk}}_{I}$)**. With *d* and $e_{I}$, judge $R_{1}\overset{?}{=}{R_{2}}^{d\cdot e_{I}}$. If the equation holds, output 1, otherwise outputs 0.

Notice that the Authority or Private Cloud holds msk and generates secret keys for users. It can help users compute Test if needed.

### 7.2. Multi-User Ranked PEKS

**Figure 8:**
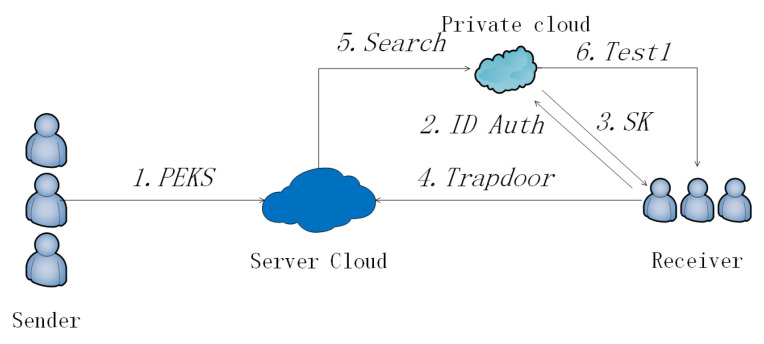
Multi-user ranked PEKS (MU-R-PEKS).

**Figure 9:**
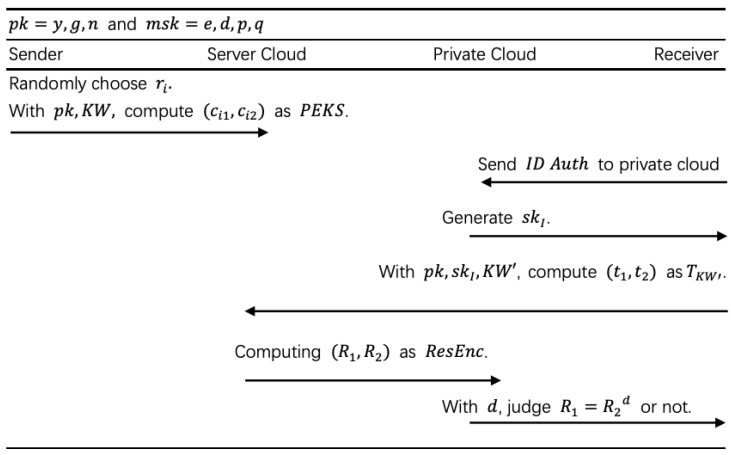
Flow of MU-R-PEKS.

In this subsection, we use our schemes to construct a multi-user ranked PEKS (Figure 8) and Figure 9 shows the detailed flow.

- **MU-R-PEKS.Setup ($1^{k}$)**. Run $R-PEKS.Setup(1^{k})$ and output y,g,n as public key pk and p,q,e,d as master secret key msk.
- **MU-R-PEKS.Secretkey ($\mathrm{pk},\mathrm{msk},{\mathrm{IDAuth}}_{I}$)**. The input of this algorithm is the system’s pk, msk and IDAuth, where IDAuth is an authority of identity. It outputs the secret key $sk_{I}$ for $Receiver_{I}$. Uniformly choose a random number $e_{I}$ such that $1<e_{I}<\phi \left(n\right)$ and $gcd(e_{I},\phi \left(n\right))=1$. Compute $d_{I}$ such that $d_{I}\cdot e_{I}=1\;mod\;\phi \left(n\right)$ with a Extended Euclidean algorithm. Output $d,e_{I}$ as $Receiver_{I}$’s $sk_{I}$ and $d_{I}$ as $Receiver_{I}$’s $pk_{I}$.
- **MU-R-PEKS.PEKS (pk,KW)**. Run R−PEKS.PEKS(pk,KW) and output the result as PEKS.
- **MU-R-PEKS.Trapdoor ($\mathrm{pk},\mathrm{msk},{\mathrm{KW}}^{\prime },{\mathrm{pk}}_{I}$)**. Run $R-PEKS.Trapdoor(pk,msk,KW^{\prime })$ and output $\left(t_{1},t_{2}\right)=\left(PEKS.t_{1},{R-PEKS.t_{2}}^{d_{I}}\right)$ with *x* as $T_{KW^{\prime }}$.
- **MU-R-PEKS.Search($\mathrm{pk},x,{\mathrm{KW}}^{\prime },{\mathrm{pk}}_{I}$)**. For any message’s PEKS, compute and output $\left(R_{1},R_{2}\right)=\left(\prod {\left(c_{i1}\right)}^{x_{i}}\cdot t_{1},{\left(\prod {\left(c_{i2}\right)}^{x_{i}}\right)}^{d_{I}}\cdot t_{2}\right)$ as ResEnc.
- **MU-R-PEKS.Test1 (pk,ResEnc,msk)**. With *e* and *q*, compute and output $R_{1}^{*}={R_{1}}^{(q-1)e}$ and $R_{2}^{*}={R_{2}}^{q-1}$ as $ResEnc^{*}$.
- MU-R-PEKS.Test2 ($\mathrm{pk},{\mathrm{ResEnc}}^{*},{\mathrm{sk}}_{I}$). With $e_{I}$, compute $R=\frac{{R_{2}^{*}}^{e_{I}}}{R_{1}^{*}}$.

To realize ranked functionality, users can finish the trapdoor generation and result test, necessarily with Authority or Private Cloud’s help. It is the designed authentication mechanism.

### 7.3. Privacy Analysis

The multi-user model is realized through a private cloud. The private cloud must be trusted which means it will always be honest. This is because the system needs an authority to manage the users. However, the privacy is still secure and cloud or any other outside people can obtain nothing about data and keywords. As the description of flows, this scheme is runs the algorithm of PEKS and ranked PEKS. The computing cost depends on PEKS and ranked PEKS. That means these are friendly with IoT devices and users.

## 8. Performance Analysis

In this section, we analyze the performance of our schemes. The notation used in this section is in Table 1. We use *e* and *m* to represent the time to compute the modular exponentiation and modular multiplication, respectively. One of our scheme’s advantage is that most computation cost is undertaken by Cloud. The rest done by users is analyzed as follows. The number of keywords for one search, the number of all keywords and the number of file are respectively denoted as $n_{k},n,$ and $n_{f}$.

Firstly, we analyze the computation cost of our schemes and verify the efficiency of our solutions.

For a Receiver of the PEKS scheme, the Receiver needs to compute 2·e and $(2n_{k}+1)\cdot m$ in the Trapdoor phase. Because it needs 1·e in Test to verify a file, the computation of Test phase is about $n_{f}\cdot e$.

For a Receiver of the ranked PEKS scheme, Receiver needs to compute $(2+n-n_{k})\cdot e$ and (3n+1)·m in Trapdoor phase. Because it needs 3·e in Test to verify a file, the computation of Test phase is about $3n_{f}\cdot e$.

For a Receiver of the MR-PEKS scheme, the Receiver needs to compute 3·e and $(2n_{k}+1)\;m$ in the Trapdoor phase. Because it needs 2·e in Test to verify a file, the computation of Test phase is about $2n_{f}\cdot e$.

For a Receiver of the MR-Ranked-PEKS scheme, the Receiver needs to compute $(3+n-n_{k})\cdot e$ and (3n+1)·m in Trapdoor phase. Because it needs 1·e in Test to verify a file, the computation of Test phase is about $n_{f}\cdot e$.

All of the above schemes’ cost is $O(n_{k},n,n_{f})$, which means that the cost of computation is acceptable. (see Table 2 and Table 3)

In order to evaluate the efficiency of schemes in experiments, we used a security parameter 1024 which is widely used in the RSA encyption. The following experiments were based on coding language Python 3.5 on macOS system(10.13.3) with an Intel(R) Core(TM) 2 Duo CPU of 2.7 GHZ and 8.00-GB RAM.

**Figure 10:**
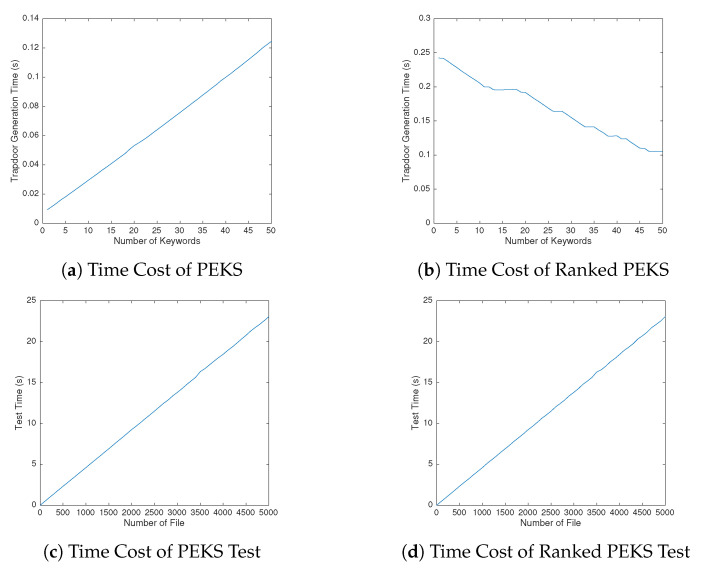
Time cost of our schemes.

We repeated the experiments 200 times and averaged the results. As shown in Figure 6, the running time of trapdoor generation is below 0.35 s. In order to get schemes with basic assumptions, we do not use any bilinear pairing. There are more complex PEKS structures in our schemes and their cost is still acceptable. As shown in Figure 10a, the cost of our PEKS scheme is almost linear growth with the number of keywords, and as shown in Figure 10b, there is a trade-off between functionality and efficiency. We also do the test of a large number of files. As a result shown in Figure 10c,d, the efficiency is just acceptable and need to be improved.

Furthermore, we compare the performance of our multi-user PEKS (MU-PEKS) scheme with related work [18,19]. In order to visualize the results easily, we ignore hash function which is faster than other operations by more than an order of magnitude. For the MU-PEKS and PEKS are not exactly the same, we maintain the similarity of the two and remove the different functions for comparison. We choose the PEKS scheme without rank and reduce attribute number parameters. The the two kinds of schemes both are transferred into a simple functional search encryption but are kept in the multi-keyword and multi-user model. In particular, if ABEKS just has one attribute in the whole system, its access control structure will be the same as ours which will only allow and deny a user. However, the efficiency of ABEKS is greatly improved by cutting functions. Before cutting functions, we summarize the computational complexity in both fully functional states as Table 4. Notice that the computing of pairings is just in ABEKS and our scheme is independent of the attribute number and access control sizing.

After ABEKS cutting access control function, a similar summary is in Table 5. Absolutely, the computing cost is much lower than before. A cloud-based IoT system may care about the computing cost of IoT equipment or sensors.

On the other side, the computing power of the cloud is usually a surplus. So, we focus on the other participants’ computing cost. Table 6 is the computational complexity of IoT devices (Sensors) and Table 7 is the computational complexity of users.

In order to evaluate the efficiency of schemes in experiments, we used a security parameter 1024. The following experiments were based on coding language Python 3.5 on macOS system(11.0.1) with an Intel(R) Core(TM) 2 Duo CPU of 2.7 GHZ and 8.00-GB RAM. The pairing experiments were based on Pairing-Based Cryptography library [23].

**Figure 11:**
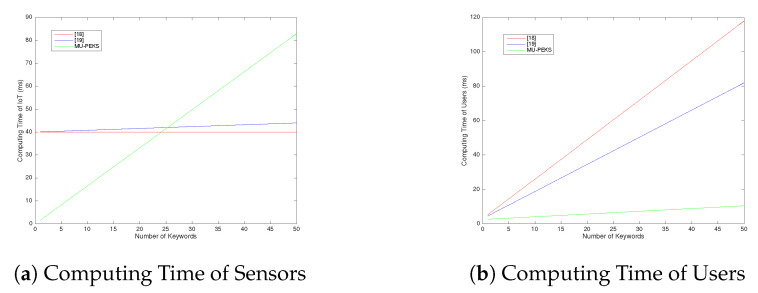
Comparison of time cost.

We repeated the experiments 200 times and averaged the results. We let $n_{l}=20$, that is just a general setting. The value of $n_{l}$ just influences the position of the intersection, but not the trend of the curve. Figure 11 shows the result. When the keyword number is small, our scheme needs less time. It means that when the task is lightweight, our scheme has an advantage. This feature is particularly suitable for Internet of Things devices (sensors). In the view of users, the whole computing cost of our MU-PEKS is less than the ABEKS Which is showed in Figure 11b.

## 9. Conclusions

In this paper, we proposed four schemes against a keyword guessing attack which is an inherent vulnerability of the traditional PEKS framework. They are proved to be secure in security model under DDH and PF assumption. We analyzed the privacy of them, and nothing about data and keywords can be leaking. In this paper, we achieve the functionality of multiple keywords search, ranked result and multi-user. Furthermore, our scheme uses only modular exponentiation and modular multiplication instead of bilinear pairing. We analyzed the computational complexity of our algorithm and finished experience to verify the performance. As a result, our schemes are friendly to the IoT system.

## Figures and Tables

**Table 1:** Notation used in performance analysis.

Notation	Description
λ	The security number
*e*	The modular exponentiation operation in group *G*
*m*	The modular multiplication operation in group *G*
$n_{k}$	The number of keywords for one search
*n*	The number of all-key words
$n_{f}$	The number of file
*p*	The bilinear pairing operation in ABEKS
$N_{a}$	The number of attribute in ABEKS
$N_{l}$	The number of leaf nodes of access structure in ABEKS

**Table 2:** The efficiency of the trapdoor phase.

Scheme	PEKS	Ranked PEKS	MU PEKS	MU Ranked PEKS
*e*	2	$2+n-n_{k}$	3	$3+n-n_{k}$
*m*	$2n_{k}+1$	3n+1	$2n_{k}+1$	3n+1

**Table 3:** The efficiency of test phase.

Scheme	PEKS	Ranked PEKS	MU PEKS	MU Ranked PEKS
*e*	$n_{f}$	$3n_{f}$	$2n_{f}$	$n_{f}$

**Table 4:** The comparison of computation complexity.

Scheme	[18]	[19]	MU PEKS
Setup (or KeyGen)	$(2n_{a}+2)\cdot e$	$(2n_{a}+2)\cdot e$	log(λ)·e
PEKS (or Encrypt)	$(2n_{l}+2)\cdot e$	$\left(2n_{l}+2\right)\cdot e+n_{k}\cdot m$	$3n_{k}\cdot e+2n_{k}\cdot m$
Trapdoor	$\left(2n_{a}+1\right)\cdot e+n_{k}\cdot log\left(n\right)\cdot m$	$\left(2n_{a}+1\right)\cdot e+n_{k}\cdot m$	$3\cdot e+2n_{k}\cdot m$
Search and Test	$\left(2n_{a}+1\right)\cdot p+\left(n_{a}+n_{k}-1\right)\cdot e$	$\left(2n_{a}+1\right)\cdot p+\left(n_{a}+n_{k}-1\right)\cdot e$	$\left(6n_{k}+2\right)\cdot e+\left(3n_{k}+3\right)\cdot m$

**Table 5:** The comparison without complex access control.

Scheme	[18]	[19]	MU PEKS
Setup (or KeyGen)	4·e	4·e	log(λ)·e
PEKS (or Encrypt)	$(2n_{l}+2)\cdot e$	$\left(2n_{l}+2\right)\cdot e+n_{k}\cdot m$	$3n_{k}\cdot e+2n_{k}\cdot m$
Trapdoor	$3\cdot e+n_{k}\cdot log\left(n\right)\cdot m$	$3\cdot e+n_{k}\cdot m$	$3\cdot e+2n_{k}\cdot m$
Search and Test	$3\cdot p+n_{k}\cdot e$	$3\cdot p+n_{k}\cdot e$	$\left(6n_{k}+2\right)\cdot e+\left(3n_{k}+3\right)\cdot m$

**Table 6:** The comparison of sensors’ computation complexity.

Scheme	[18]	[19]	MU PEKS
PEKS (or Encrypt)	$(2n_{l}+2)\cdot e$	$\left(2n_{l}+2\right)\cdot e+n_{k}\cdot m$	$3n_{k}\cdot e+2n_{k}\cdot m$

**Table 7:** The comparison of users’ computation.

Scheme	[18]	[19]	MU PEKS
Trapdoor	$3\cdot e+n_{k}\cdot log\left(n\right)\cdot m$	$3\cdot e+n_{k}\cdot m$	$3\cdot e+2n_{k}\cdot m$
Search and Test	$3\cdot p+n_{k}\cdot e$	$3\cdot p+n_{k}\cdot e$	$\left(6n_{k}+2\right)\cdot e+\left(3n_{k}+3\right)\cdot m$
Total	$3\cdot p+\left(3+n_{k}\right)\cdot e+n_{k}\cdot log\left(n\right)\cdot m$	$3\cdot p+\left(3+n_{k}\right)\cdot e+n_{k}\cdot m$	$5\cdot e+2n_{k}\cdot m$

## Abbreviations

The following abbreviations are used in this manuscript:

IoT	Internet of Things
PEKS	Public-key Encryption with Keyword search
SSE	Searchable Symmetrical Encryption
PIR	Private Information Retrieval
IND-CCA	Indistinguishability under chosen-ciphertext-attack
ABE	Attribute-based encryption
ABEKS	Attribute-based encryption with keyword search
SME	Secure Match Encryption
KGA	Keyword guessing attack
DH	Diffie–Hellman
DDH	Decisional Diffie–Hellman assumption
PF	Prime factorization assumption
PPT	Probabilistic polynomial time
R-PEKS	Ranked public-key encryption with keyword search
MU-PEKS	multi-user public-key encryption with key word search
MU-R-PEKS	Multi-user ranked public-key encryption with key word search
MDPI	Multidisciplinary Digital Publishing Institute
DOAJ	Directory of open access journals
TLA	Three letter acronym
LD	linear dichroism
